# *Toxoplasma* Shelph, a Phosphatase Located in the Parasite Endoplasmic Reticulum, Is Required for Parasite Virulence

**DOI:** 10.1128/msphere.00350-22

**Published:** 2022-11-03

**Authors:** Rania Najm, Maguy Hamie, Laurence Berry-Sterkers, Maryse Lebrun, Hiba El Hajj, Mauld H. Lamarque

**Affiliations:** a Department of Biomedical Sciences, College of Medicine, Mohammed Bin Rashid University of Medicine and Health Sciences, Dubai, United Arab Emirates; b Department of Experimental Pathology, Immunology and Microbiology, American University of Beirut, Beirut, Lebanon; c LPHI, UMR 5235 CNRS, Université de Montpellier, Montpellier, France; University of Georgia

**Keywords:** Toxoplasma gondii, enzyme kinetics, infectious disease, parasitology, reverse genetic analysis, serine/threonine phosphatases, subcellular localization

## Abstract

Toxoplasma gondii is a single-celled parasitic eukaryote that evolved to successfully propagate in any nucleated cell. As with any other eukaryote, its life cycle is regulated by signaling pathways controlled by kinases and phosphatases. T. gondii encodes an atypical bacterial-like phosphatase absent from mammalian genomes, named Shelph, after its first identification in the psychrophilic bacterium *Schewanella* sp. Here, we demonstrate that *Toxoplasma* Shelph is an active phosphatase localized in the parasite endoplasmic reticulum. The phenotyping of a *shelph* knockout (KO) line showed a minor impairment in invasion on human fibroblasts, while the other steps of the parasite lytic cycle were not affected. In contrast with *Plasmodium* ortholog Shelph1, this invasion deficiency was not correlated with any default in the biogenesis of secretory organelles. However, Shelph-KO parasites displayed a much-pronounced defect in virulence *in vivo*. These phenotypes could be rescued by genetic complementation, thus supporting an important function for Shelph in the context of a natural infection.

**IMPORTANCE** Toxoplasma gondii belongs to the *Apicomplexa* phylum, which comprises more than 5,000 species, among which is Plasmodium falciparum, the notorious agent of human malaria. Intriguingly, the *Apicomplexa* genomes encode at least one phosphatase closely related to the bacterial *Schewanella* phosphatase, or Shelph. To better understand the importance of these atypical bacterial enzymes in eukaryotic parasites, we undertook the functional characterization of T. gondii Shelph. Our results uncovered its subcellular localization and its enzymatic activity, revealed its subtle involvement during the tachyzoite invasion step of the lytic cycle, and more importantly, highlighted a critical requirement of this phosphatase for parasite propagation in mice. Overall, this study revealed an unexpected role for T. gondii Shelph in the maintenance of parasite virulence *in vivo*.

## INTRODUCTION

*Apicomplexa* parasites are pathogens of human and veterinary importance, including *Plasmodium* spp., the etiologic agent of malaria, and Toxoplasma gondii, which is responsible for toxoplasmosis. These obligate intracellular parasites display complex life cycles alternating between definitive and intermediate hosts, such as humans. Upon infection by T. gondii via the ingestion of cysts or sporulated oocysts shed in cat feces, the parasites invade enterocytes and differentiate into fast-replicating forms called tachyzoites (1). Upon invasion, they establish a surrounding vacuole within the host cell and start replicating in this niche. At the end of the multiplication process, the newly formed daughter cells egress from the host and can initiate another cycle. The iteration of this lytic cycle causes massive cellular destruction and simultaneously triggers a proinflammatory host response (2–4). It is believed that under the pressure of the immune system, the parasite converts into slow-replicating dormant stages called bradyzoites that form tissue cysts primarily in the brain and skeletal muscles (5). These cysts persist for the lifetime of the host and can reactivate into tachyzoites in immunocompromised patients.

The lytic cycle of T. gondii relies on reversible phosphorylation ensured by parasite kinase homologs to mammalian enzymes, such as protein kinase G (PKG) or protein kinase A (PKA), which regulate egress (6–11), replication (11, 12), invasion (13), and the transition from tachyzoite to bradyzoite (14–16). In addition to these well-conserved kinases, *Apicomplexa* parasites possess an expanded repertoire of plant-like kinases named calcium-dependent kinases (CDPKs) (17), of which CDPK1 and -3 have been shown to be involved in invasion and egress, respectively (18–20). In contrast to the wealth of data highlighting the importance of parasite kinases, the functions of most parasite phosphatases remains unexplored. Classically, phosphatases are subdivided into three groups: the protein phosphatases (PPP), the metallo-dependent phosphatases (PPM), and the tyrosine phosphatases (PTP) (21), the former two being serine/threonine phosphatases. The PPP family is likely very ancient, as members can be found in prokaryotes and eukaryotes, and it is further subdivided into PP1, PP2A, PP2B, PP4, PP5, PP6, PP7, and PPKL enzymes. A bioinformatic search performed on the T. gondii genome identified 11 PPPs and 33 PPMs (22). Among the PPP family, *Apicomplexa* genomes, with the exception of Babesia bovis, encode bacterial-like phosphatases that are absent from the human genome but found in plants and a red alga, Porphyra yezoensis (22, 23). Because of their similarity with a phosphatase of the psychrophilic bacteria *Schewanella* spp., they are named Schewanella-like phosphatases, or Shelph (Slp). In Arabidopsis thaliana, *At*Slp1 and -2 display chloroplast and mitochondrial localization, respectively, with *At*Shl2 shown to regulate seed germination (24, 25). The *Plasmodium* spp. genome encodes two Slps, annotated Slp1 and Slp2, that display tyrosine phosphatase activity despite harboring most of the conserved residues found in the PPP subgroup (26, 27). In Plasmodium berghei, a rodent model of *Plasmodium* spp., *Pb*Slp1 preferentially localizes to the endoplasmic reticulum (ER) and is associated with membrane fractions (27). Although dispensable for asexual development and gametocytogenesis in red blood cells, *Pb*Slp1 knockout (KO) parasites were impaired in ookinete conversion *in vitro* and were unable to form oocysts in the mosquito midgut epithelium (27). Ultrastructural analysis of *Pb*Slp1-KO ookinetes revealed a major defect in apical microneme formation, suggesting a potential role for *Pb*Slp1 in microneme maturation and/or trafficking of micronemal proteins. In Plasmodium falciparum, *Pf*Slp2 is a late-expressed protein during the erythrocytic cycle, is apically localized in merozoites, and is likely secreted in the host at the time of invasion (26, 28). However, *Pf*Slp2-KO parasites did not exhibit any defect during asexual development, suggesting that its function may be either compensated by another phosphatase or relevant in other stages of the P. falciparum life cycle.

Among the 44 serine/threonine phosphatases identified *in silico* in the T. gondii genome, only one Slp protein was identified (22). A recent functional study performed on 17 phosphatases of T. gondii reported Slp localization in the cytoplasm and dense granules of the parasite (29). In addition, deletion of the *slp* gene did not impact the parasite lytic cycle in human fibroblasts or its virulence in mice.

In this study, we investigated the localization, enzymatic activity, and function of *Tg*Slp in tachyzoites. We found that *Tg*Slp is an active phosphatase dispensable to complete the lytic cycle *in vitro*. However, in contrast to the published data, our results support an ER localization for *Tg*Slp and indicate that the virulence of Slp-KO parasites is strongly attenuated in mice. Importantly, this phenotype can be rescued by genetic complementation. Altogether, our results suggest that the phosphatase fulfills an important function for parasite survival *in vivo*.

## RESULTS

### TGME49_254770 is a putative Schewanella-like phosphatase.

T. gondii genome analysis revealed the presence of one Schewanella-like phosphatase ortholog (22), TGME49_254770, that we renamed *Tg*Slp throughout the study. Protein alignment with P. falciparum Slp1 and Slp2 indicated that *Tg*Slp shares 32% identity with *Pf*Slp1, versus 28% with *Pf*Slp2 (see Fig. S1A and B in the supplemental material). As previously described for *Pf*Slp, *Tg*Slp displays a conserved central metallo-phosphatase domain (amino acids 95 to 321) exhibiting signature motifs characteristic of the PPP family (GDXHG, GDXVDRG, and GNH[E/D]), as well as specific residues involved in metal ion coordination (Fig. S1B), suggesting that the protein is likely an active phosphatase. No signal peptide was predicted in the *Tg*Slp coding sequence, but a putative transmembrane domain was sometimes predicted between amino acids 57 and 77, depending on the software used.

10.1128/msphere.00350-22.1 FIG S1 Homology between P. falciparum Slp1 and Slp2 and T. gondii Slp. (A) Percent identity between *Pf*Slp1, *Pf*Slp2, and *Tg*Slp at the protein level, based on BLAST analysis. (B) Primary amino acid alignment of P. falciparum Slp1 and Slp2 and T. gondii Slp. Conserved or similar residues are highlighted in black and gray, respectively. Alignment was performed using Clustal O and imported into BoxShade for image assembly. Orange boxed amino acids correspond to highly conserved motifs comprising the active site of canonical serine/threonine phosphatases of the phosphoprotein phosphatases (PPP) family (GDXHG, GDXXDRG, GNH[E/D], and H[A/G]G), which contain most of the residues coordinating metal ion binding (orange ball). The amino acids highlighted in yellow correspond to the C-terminal GDEL motif, which resembles the eukaryote ER targeting signal. Download FIG S1, TIF file, 0.5 MB. Copyright © 2022 Najm et al. 2022 Najm et al. https://creativecommons.org/licenses/by/4.0/ This content is distributed under the terms of the Creative Commons Attribution 4.0 International license.

### *Tg*Slp is primarily located in the endoplasmic reticulum.

**FIG 1:**
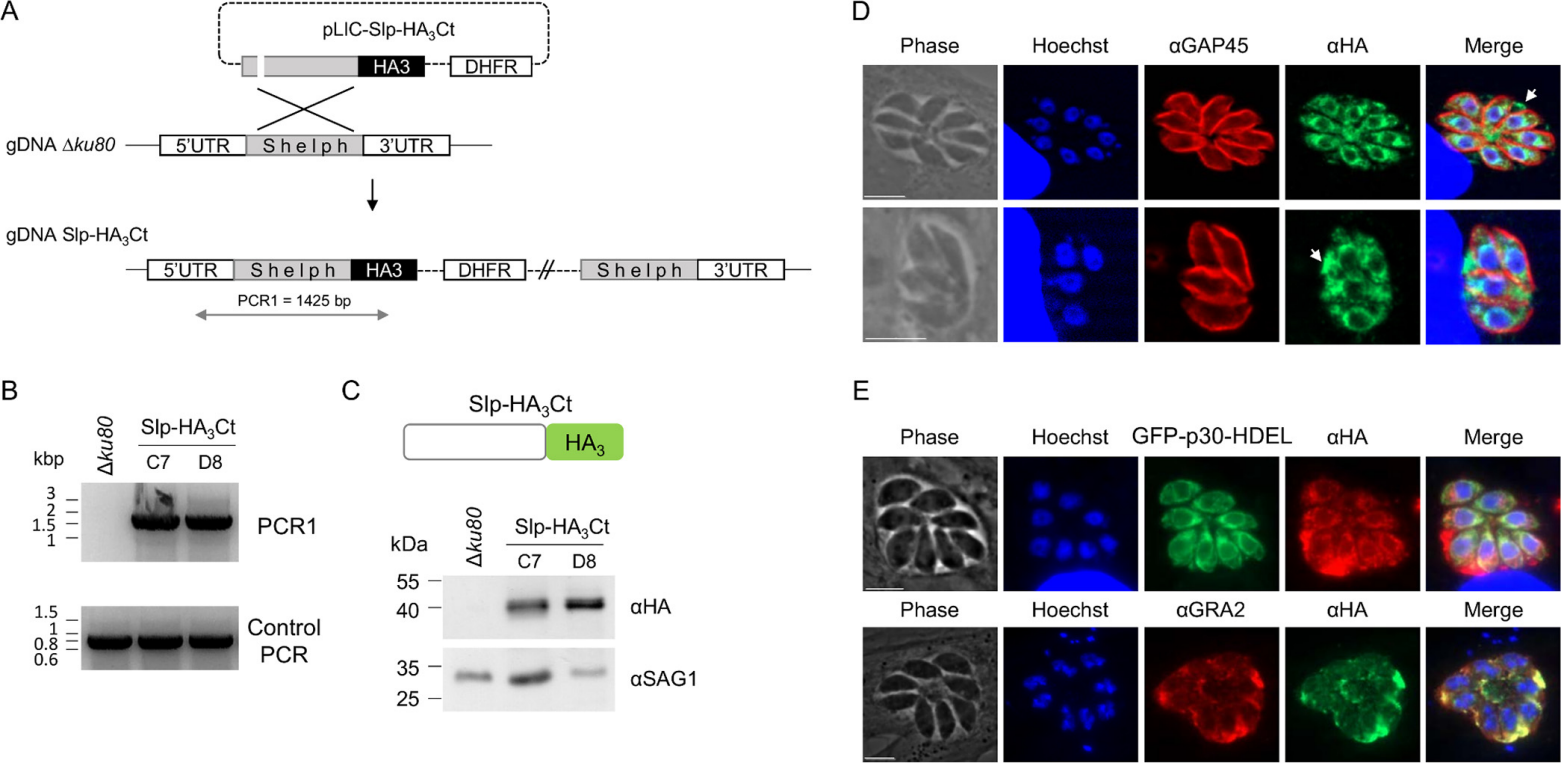
Slp-HA_3_Ct behaves as a dense granule protein. (A) Scheme depicting the single-crossover integration of pLIC-Slp-HA_3_Ct at the endogenus *slp* locus. PCR1 corresponds to the integrative PCR shown in panel B. (Size of the amplicon is in base pairs.) (B) Integrative PCR for pLIC-Slp-HA_3_Ct genomic integration (PCR1) showing the expected 1,425-bp amplification for clones C7 and D8. The control PCR corresponds to the amplification of *ron10*. (C, top) The scheme represents Slp-HA_3_Ct protein with the C-terminal location of the HA_3_ tag. (Bottom) Immunoblot of Slp-HA_3_Ct lines versus the parental Δ*ku80* using anti-HA antibodies. SAG1, loading control. (D) IFA of Slp-HA_3_Ct parasites using anti-HA antibodies showing the localization of Slp-HA_3_ protein in close proximity to the nucleus but also secreted in the PV (white arrows). GAP45, inner membrane complex marker. Nuclei were stained with Hoechst. Scale bar, 5 μm. (E) IFA of Slp-HA_3_Ct parasites using anti-HA and either GFP-p30-HDEL as an ER marker or anti-GRA2 antibodies, showing a large colocalization of the proteins in the ER and the PV lumen, respectively. Nuclei were stained with Hoechst. Scale bar, 5 μm.

**FIG 2:**
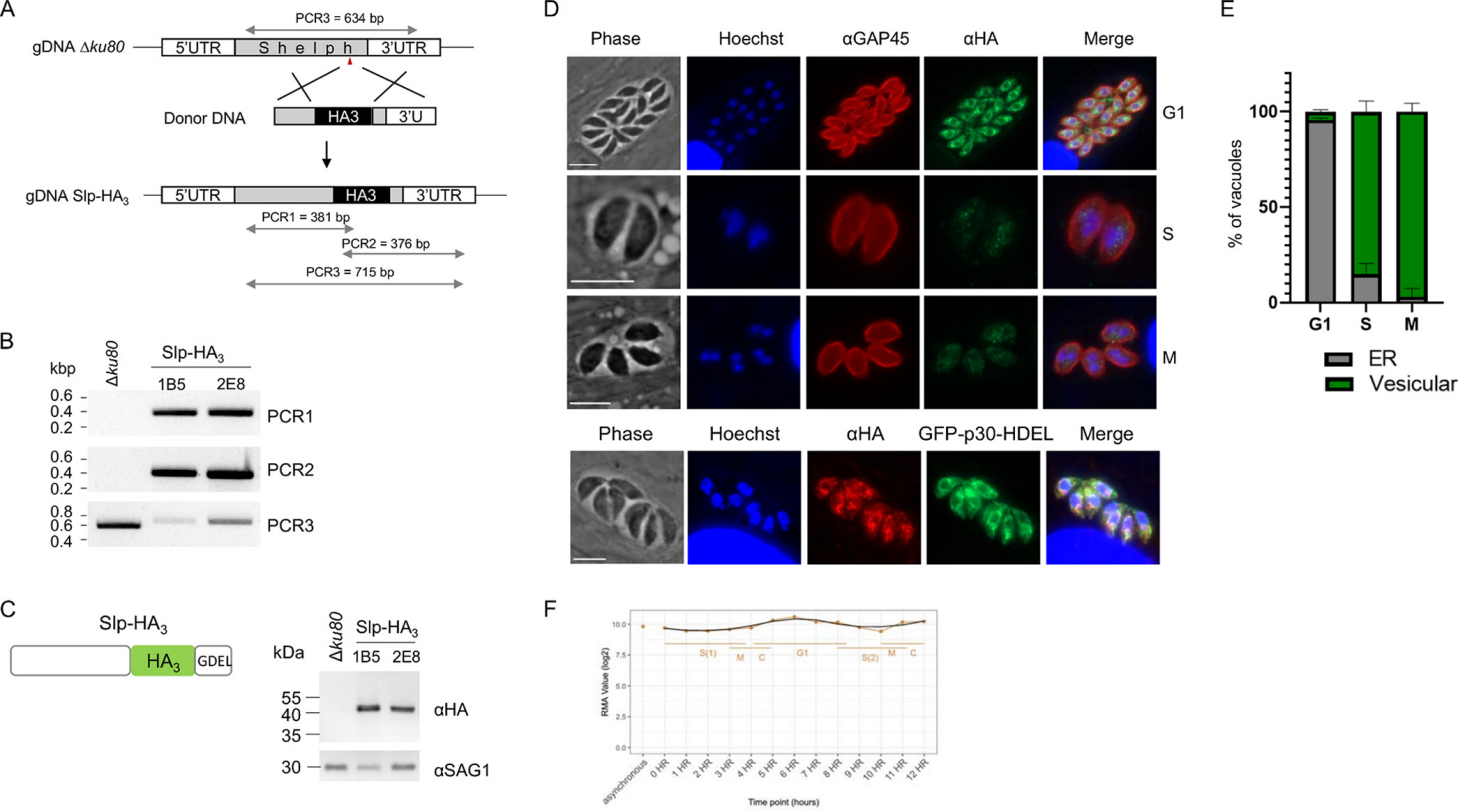
Slp-HA_3_ is an ER-resident protein. (A) Scheme showing the 2 homology regions used to insert an HA_3_ tag 9 residues before GDEL using CRISPR-Cas9. The red arrow corresponds to the guide RNA sequence. The diagnostic PCRs used in panel B are shown below the edited genomic DNA, and the sizes of the expected amplicons are indicated in base pairs. (B) Diagnostic PCRs confirming the successful modification of the genome, performed on clones 1B5 and 2E8, as depicted in panel A. (C, left) The scheme represents Slp-HA_3_ protein with the location of the HA_3_ tag 9 amino acids before the C-terminal GDEL motif. (Right) Immunoblot of Δ*ku80* or Slp-HA_3_ parasites using anti-HA antibodies. SAG1, loading control. (D) IFA of Slp-HA_3_ parasites using anti-HA and anti-GAP45 antibodies or anti-HA alone on parasites expressing the ER marker GFP-p30-HDEL. Hoechst staining and phase were used to monitor the phase of the cell cycle (G_1_, S, or M). Scale bar, 5 μm. (E) Quantification of the distribution of Slp-HA_3_ localization along the parasite cell cycle. The graph represents means of 2 quantifications ± standard deviation. Number of vacuoles observed in G_1_, S, and M (*n* = 323, 78, and 36, respectively). (F) T. gondii RH cell cycle microarray expression profile of *Tgslp* (45). Robust multi-array average (RMA) normalized values are shown (base log_2_).

We first investigated the subcellular localization of Slp by using a ligation-independent cloning (LIC) system to add a C-terminal triple hemagglutinin tag (HA_3_) at the endogenous locus by single crossover in the Δ*ku80* background (Fig. 1A). Diagnostic PCRs performed on two clones (C7 and D8) confirmed the correct integration of the plasmid (Fig. 1B), and the resulting fusion protein, named Slp-HA_3_Ct for C-terminal tagging, was detected by Western blotting as a single band of the expected molecular mass (51 kDa) (Fig. 1C). In an immunofluorescence assay (IFA), the majority of the parasites exhibited a perinuclear labeling that partially colocalized with the ER reporter GFP-p30-HDEL (Fig. 1D and E). In addition, a strong fluorescent signal was also detected in the parasitophorous vacuole (PV), which colocalized with the dense granule protein GRA2 (Fig. 1D and E), supporting *Tg*Slp as a dense granule protein, as previously reported (29). However, we noticed a C-terminal GDEL motif in the protein sequence at positions 417 to 420 (Fig. S1B), reminiscent of the canonical ER retention signal H/KDEL for soluble protein retrieval to the ER (30, 31). We hypothesized that the HA_3_ C-terminal tagging of the protein perturbed the retrieval of Slp to the ER, which as a consequence may have favored its secretion in the PV. To investigate this possibility, we used CRISPR-Cas9 technology to introduce an HA_3_ tag 9 amino acids before the GDEL motif (Fig. 2A). The strain thereby obtained, named Slp-HA_3_, was verified by PCR and sequencing (Fig. 2B; Fig. S2), and the fusion protein was correctly expressed (Fig. 2C). In the IFA, the protein appeared again, staining in proximity with the nucleus that largely colocalized with the ER reporter GFP-p30-HDEL (Fig. 2D), further supporting an ER localization. Importantly, Slp-HA_3_ was no longer detected in the PV, thereby indicating that a free C terminus is important for the proper targeting of the protein to the ER. In addition, Slp-HA_3_ expression oscillated along the cell cycle, with parasites in G_1_ phase exhibiting the strongest signal while those in S and M phases showed a very weak vesicular staining in the parasite cytoplasm (Fig. 2D and E). This timing of expression was in agreement with the available transcriptomic data on *Tgslp* (32) (Fig. 2F). Altogether, our results indicated that *Tg*Slp is mainly localized in the ER of the parasite, which is also concordant with the recent spatial assignment of Slp by hyperLOPIT (33).

10.1128/msphere.00350-22.2 FIG S2 Sequencing of the *slp-HA_3_* locus. The image corresponds to the alignment of the nucleotidic sequences of *slp* wild type and *slp-HA3* loci, obtained with the software Geneious. The corresponding amino acids are shown below the nucleotide sequences. The sequencing confirmed the insertion of the HA_3_ tag (highlighted in green), 9 amino acids upstream of the C-terminal GDEL motif (highlighted in yellow). Download FIG S2, TIF file, 0.7 MB. Copyright © 2022 Najm et al. 2022 Najm et al. https://creativecommons.org/licenses/by/4.0/ This content is distributed under the terms of the Creative Commons Attribution 4.0 International license.

### Slp is a bona fide phosphatase.

**FIG 3:**
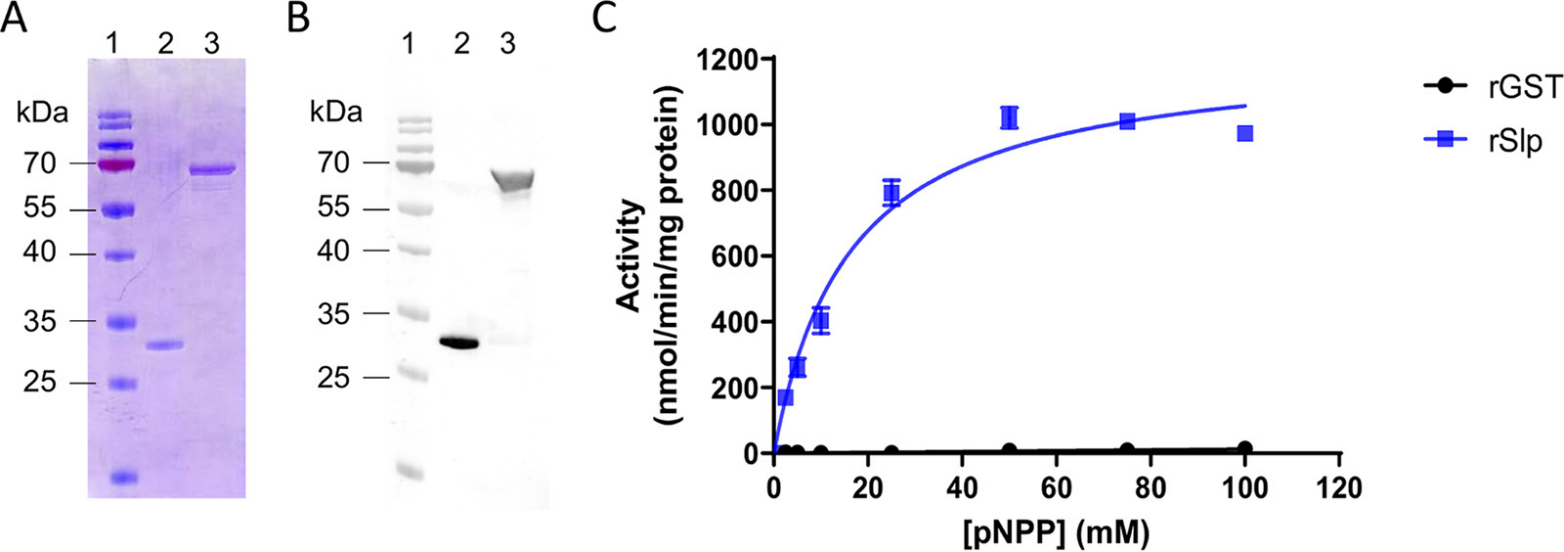
Recombinant rSlp is an active phosphatase. (A) SDS-PAGE of rGST (lane 2) or rSlp (lane 3) stained with Coomassie blue. Lane 1, page ruler prestained protein ladder. (B) Immunoblot of the same proteins as in panel A revealed with anti-GST antibodies. (C) Fitted curve of the phosphatase activity of rGST or rSlp against increasing concentrations of pNPP (*n* = 4 independent experiments, with means ± SEM shown).

To verify the predicted enzymatic activity of Slp phosphatase, we expressed a recombinant Slp protein in Escherichia coli, fused at its N terminus to a glutathione *S*-transferase (GST) tag (rSlp). Following affinity purification on glutathione-agarose beads of GST alone (rGST) or rSlp proteins (Fig. 3A and B), the phosphatase activities of the proteins were tested against the artificial substrate *para*-nitrophenylphosphate (pNPP) (Fig. 3C). In contrast to rGST, rSlp exhibited phosphatase activity toward pNPP with a calculated *V*_max_ of ~1,228 ± 37.6 nmol pNP/min/mg of protein (mean ± standard error of the mean [SEM]). This result was in agreement with the range of activities reported in other organisms (26, 34).

### Slp is dispensable *in vitro* in human fibroblast cultures.

**FIG 4:**
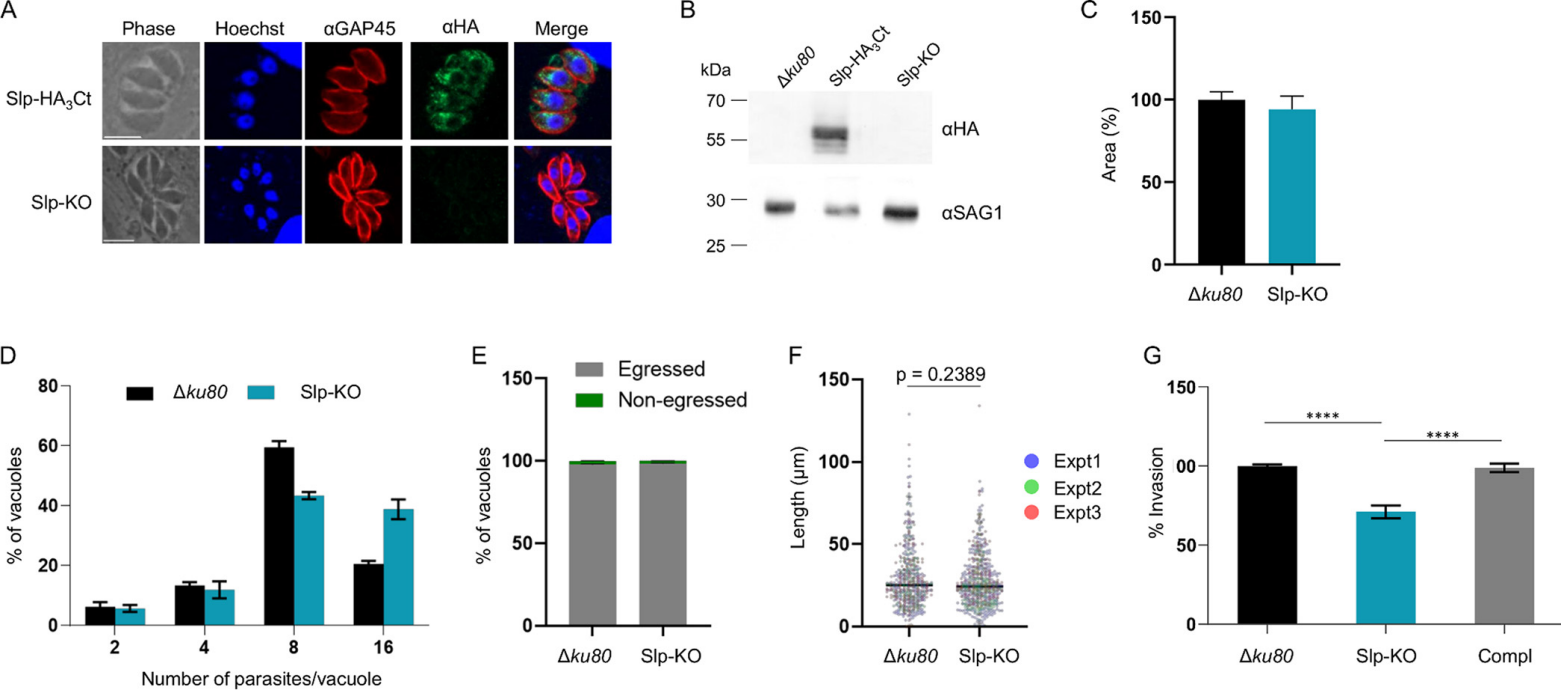
Slp-KO parasites exhibit a minor defect in invasion *in vitro*. (A) IFA of Slp-HA_3_Ct or Slp-KO parasites using anti-HA and anti-GAP45 antibodies. Nuclei were stained with Hoechst. Scale bar, 5 μm. (B) Immunoblot of Δ*ku80*, Slp-HA_3_Ct, or Slp-KO parasites using anti-HA antibodies. SAG1, loading control. (C) Plaque assays performed on Δ*ku80* or Slp-KO parasites. The plaque areas are expressed as percentages of the control cell line (*n* = 3 independent experiments; means ± SEM). (D) Replication assays performed on Δ*ku80* or Slp-KO parasites (*n* = 4 independent experiments; means ± SEM). (E) Egress assay performed on Δ*ku80* or Slp-KO parasites. Data are expressed as the percentage of vacuoles that egressed or not. The graph shows one representative experiment of two and represents the means of the experimental triplicates ± SEM. (F) Motility assay performed on Δ*ku80* or Slp-KO parasites. The graph shows the distribution of the trail lengths in 3 independent experiments. The bar represents the median, and the *P* value of a Mann-Whitney test is shown. (G) Invasion assay performed on Δ*ku80*, Slp-KO, or Compl lines (*n* = 5 independent experiments; mean ± SEM). ****, *P* ≤ 0.0001, Mann-Whitney test.

To further investigate the function of Slp, we engineered a knockout line in the Slp-HA_3_Ct background, using CRISPR-Cas9. Donor DNA consisted in the PCR amplification of the selectable *HXGPRT* cassette, flanked on both sides by 30-bp homology regions corresponding to *slp* 5′ and 3′ untranslated regions (UTRs), respectively (Fig. S3A). Edited parasites were screened by IFA for the loss of the HA signal (Fig. 4A), and the obtention of a Slp-KO line was confirmed by diagnostic PCRs and immunoblotting (Fig. S3B; Fig. 4B). In plaque assays, the Slp-KO lytic cycle was undistinguishable from that of control parasites (Fig. 4C), in agreement with a previous whole-genome screen by CRISPR-Cas9 of T. gondii tachyzoites during *in vitro* growth in fibroblasts (35). As plaque assays recapitulate several steps of the T. gondii lytic cycle in a single assay, slight defects may be overlooked, which prompted us to assess individually each step of the lytic cycle. The overall multiplication rate of Slp-KO was slightly faster than that of the parental line, with a consistently higher number of vacuoles containing 16 parasites (Fig. 4D). Slp-KO egress and gliding motility were undistinguishable from those observed in the parental line (Fig. 4E and F), while we noticed a moderate but reproducible decrease of the invasion capacities of the parasites by 20% (Fig. 4G). To ascertain whether the invasion defect was directly correlated with the loss of Slp, we complemented the Slp-KO line with the PSBLE35 cosmid (36), encompassing the *slp* locus (Compl line). PCR and reverse transcription-PCR (RT-PCR) confirmed the restoration of the endogenous *slp* locus and of *slp* transcript expression, respectively (Fig. S4A to C). Importantly, adding back the *slp* gene restored the full invasiveness of the parasite (Fig. 4G), thereby confirming that Slp phosphatase plays a role in invasion.

10.1128/msphere.00350-22.3 FIG S3 Obtention of Slp-KO parasites. (A) Scheme showing the *HXGPRT* cassette flanked by 30 bp of homology to the *slp* locus (gray arrows, primers MLa93 and MLa100), used to replace the full *slp* genomic locus and enabling the obtention of the Slp-KO parasite line. The sequence of the gRNA is shown as a red triangle. The diagnostic PCRs performed for panel B, confirming the integration, are depicted as PCRs 1 to 4. (B) Diagnostic PCRs confirming the replacement of the *slp* locus by the *HXGPRT* cassette, using PCRs 1 to 4, as shown in panel A. Control corresponds to the amplification of the *ron10* gene. Download FIG S3, TIF file, 0.2 MB. Copyright © 2022 Najm et al. 2022 Najm et al. https://creativecommons.org/licenses/by/4.0/ This content is distributed under the terms of the Creative Commons Attribution 4.0 International license.

10.1128/msphere.00350-22.4 FIG S4 Complementation of Slp-KO parasites with a cosmid. (A) Scheme depicting the *slp* loci of Δ*ku80*, Compl, or Slp-KO parasites. The PCRs used to verify the restoration of a wild-type *slp* locus in the Compl line are shown with arrows and correspond to the PCRs described in panel B. (B) PCR amplification of *slp* coding sequence (top) or the entire *slp* locus (bottom), showing the restoration of a wild-type *slp* locus in the complemented line. The expected size for the *slp* coding DNA sequence (CDS) was 1,500 bp. The expected sizes for the *slp* locus were 1,814 bp for Δ*ku80* and Compl lines and 2,398 bp for the Slp-KO line. (C) RT-PCR performed on *slp* mRNA. As there was no difference in size between *slp* mRNA and genomic DNA (gDNA), control RT-PCRs were also performed on *zfp2* mRNA (200 bp for cDNA, 400 bp for gDNA) and negative control RT-PCRs (neg) corresponding to RT reactions without the addition of reverse transcriptase, were used as a control for gDNA contamination. Download FIG S4, TIF file, 0.3 MB. Copyright © 2022 Najm et al. 2022 Najm et al. https://creativecommons.org/licenses/by/4.0/ This content is distributed under the terms of the Creative Commons Attribution 4.0 International license.

### Slp is not involved in microneme biogenesis.

**FIG 5:**
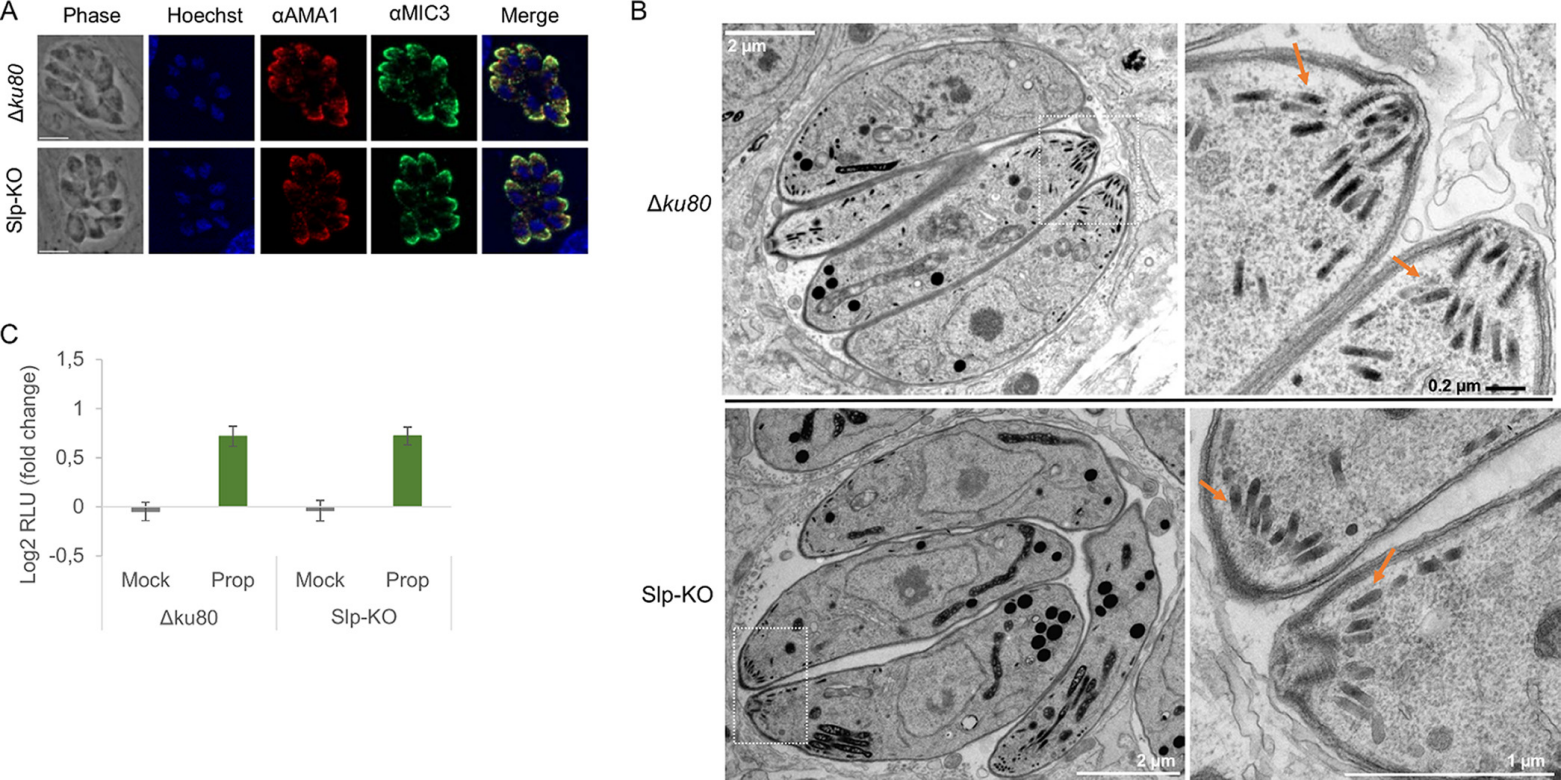
Slp-KO parasites are not impaired in microneme biogenesis. (A) IFA of the micronemes in Δ*ku80* or Slp-KO parasites using anti-AMA1 and anti-MIC3 antibodies. Nuclei were stained with Hoechst. Scale bar, 5 μm. (B) Left: TEM of Δ*ku80* (top) or Slp-KO intracellular tachyzoites (bottom) showing the region zoomed in as a dotted rectangle. Scale bar is indicated in each image. Right: Zoom of the apex of the tachyzoites. The orange arrows point to the micronemes. Scale bar, 1 μm or 0.2 μm. These are representative images of *n* = 50 for Δ*ku80* and *n* = 80 for Slp-KO. (C) Microneme secretion assay performed on Δ*ku80* or Slp-KO parasites expressing a MIC2-Gluc reporter, thereby enabling measurement of the RLU released in the medium upon microneme secretion. Data are expressed as the log_2_ fold change of the RLU measured between untreated versus treated parasites with mock treatment or propranolol (prop) (*n* = 3 independent experiments; means ± SEM).

In P. berghei, *Pb*Slp1-KO displayed a defect in zygote-to-ookinete conversion in the mosquito midgut, with a 60% reduction in ookinete formation (27). In addition, the ookinetes that could differentiate had either no apical micronemes or a reduced number of micronemes not apically located, suggesting that *Pb*Slp1 may play a role in microneme biogenesis at the ookinete stage. The similar localization of *Tg*Slp at the ER and the small defect of Slp-KO parasites in invasion prompted us to investigate carefully the formation of micronemes and their secretion in Slp-KO parasites. As two distinct micronemal proteins subsets have been described in T. gondii, based on their dependence on functional Rab5A and Rab5C for their correct microneme localization (37), we first immunolocalized AMA1 and MIC3 as representative proteins of these two subsets. Both microneme subsets decorated the apex of T. gondii tachyzoites, regardless of the presence or absence of Slp (Fig. 5A), and no obvious defect in the number of micronemes or in their apical localization was observed by transmission electron microscopy (TEM) (Fig. 5B). To analyze the ability of the mutant to secrete the content of micronemes, we opted for a quantitative assay using a MIC2-Gaussia luciferase (MIC2-GLuc) reporter (6). Hence, we expressed MIC2-GLuc at the *uprt* locus of Δ*ku80* or Slp-KO parasites (Fig. S5A). The reporter-expressing strains were verified by diagnostic PCRs (Fig. S5B). We used propranolol, an inhibitor of phosphatidic acid phosphatase, as an inducer of microneme secretion, to increase the concentration of phosphatidic acid, thereby promoting the anchoring of micronemes to the plasma membrane in an acylated pleckstrin homology domain-dependent manner (38). There was no difference in the secretion of micronemes between the control and the Slp-KO lines, further confirming that the absence of Slp does not lead to microneme defects in T. gondii (Fig. 5C). Similarly, we did not find evidence of any obvious deficiency regarding the rhoptries or dense granules by IFA or TEM (Fig. S6A to C).

10.1128/msphere.00350-22.5 FIG S5 Generation of parasites encoding the MIC2-Gluc reporter. (A) Scheme depicting the integration of a MIC2Gluc reporter at the *uprt* locus via double-crossover recombination in the 5′- and 3′-UTR of the *uprt* gene. The orange arrowheads indicate the sequences corresponding to the guide RNAs. PCR results to check the construct integration are shown as PCR1 and -2. (B) Diagnostic PCRs 1 and 2 performed on Δ*ku80*, Δ*ku80*/MIC2Gluc, or Slp-KO/MIC2Gluc parasite lines. Download FIG S5, TIF file, 0.2 MB. Copyright © 2022 Najm et al. 2022 Najm et al. https://creativecommons.org/licenses/by/4.0/ This content is distributed under the terms of the Creative Commons Attribution 4.0 International license.

10.1128/msphere.00350-22.6 FIG S6 Secretory organelle integrity in Slp-KO parasites. (A and B) IFA of the rhoptry (A) or dense granule (B) proteins in Slp-KO parasites. Hoechst, nuclei staining. Scale bar, 5 μm. (C) (Left) TEM of Δ*ku80* (top) or Slp-KO intracellular tachyzoites (bottom), showing the region zoomed in as a dotted rectangle. (Right) Enlargement of the left image, showing the proper apical positioning of the rhoptries. Download FIG S6, TIF file, 1.9 MB. Copyright © 2022 Najm et al. 2022 Najm et al. https://creativecommons.org/licenses/by/4.0/ This content is distributed under the terms of the Creative Commons Attribution 4.0 International license.

### Slp-KO parasites are impaired in virulence *in vivo*.

**FIG 6:**
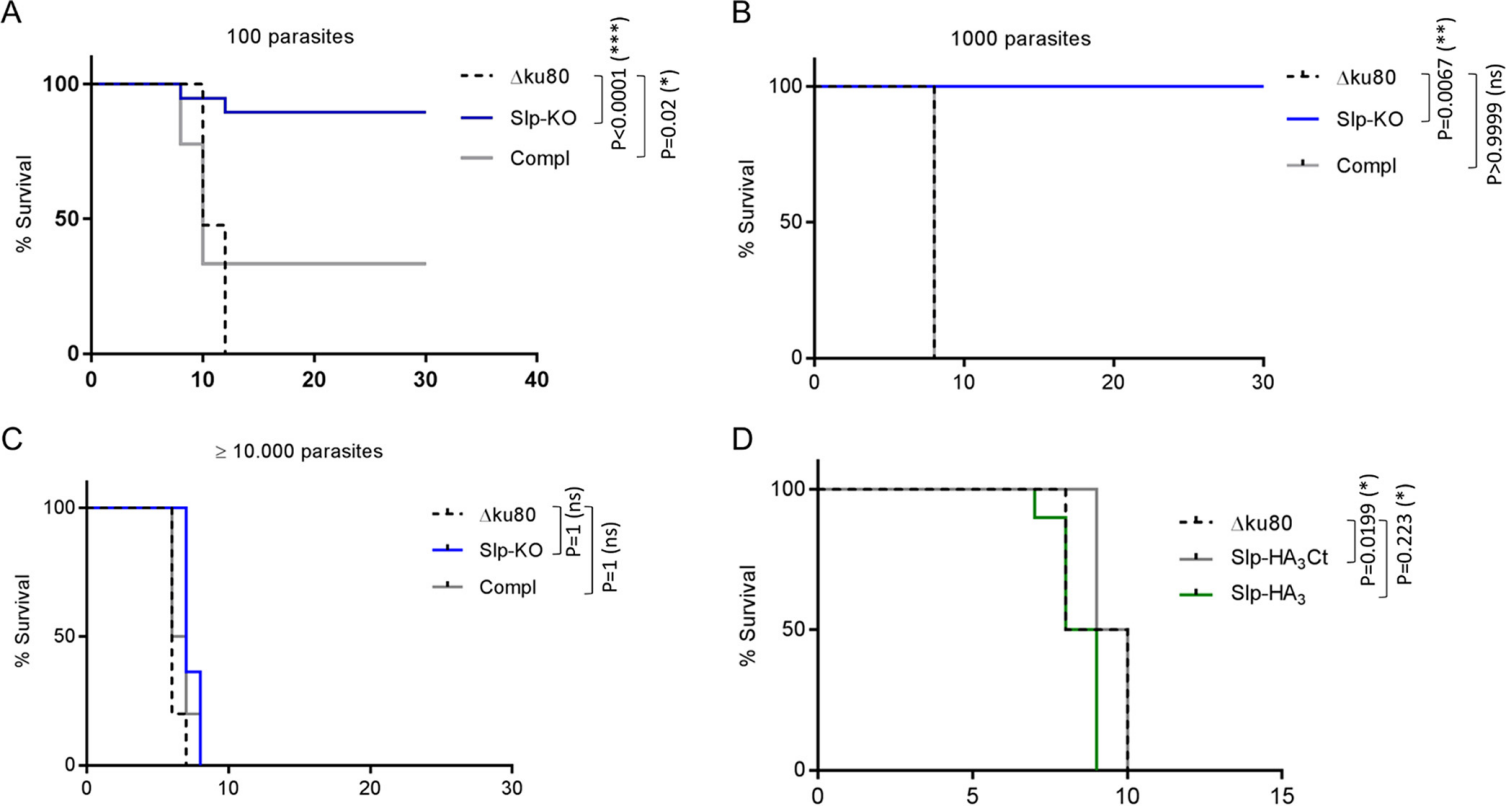
Slp-KO parasites are strongly attenuated *in vivo*. (A to C) Survival of BALB/c mice infected with 100 (A), 1,000 (B), or 10,000 (C) tachyzoites of Δ*ku80*, Slp-KO, or Compl parasite lines, respectively. The graphs represent the additive results of 4 (A) or 2 (B and C) independent experiments. Experimental groups: (A) 21 Δ*ku80* mice, 19 Slp-KO mice, 9 Compl mice; (B) 10 Δ*ku80* mice, 10 Slp-KO mice, and 10 Compl mice; (C) 12 Δ*ku80* mice, 12 Slp-KO mice, 10 Compl mice. (D) Survival of BALB/c mice infected with 100 tachyzoites of Δ*ku80*, Slp-HA_3_Ct, or Slp-HA_3_ parasite lines. The graph represents the additive results of 2 independent experiments (Δ*ku80*, 10 mice; Slp-HA_3_Ct, 10 mice; Slp-HA_3_, 10 mice).

Many genes encoded in the T. gondii genome are not essential for *in vitro* growth but yet are important for parasite survival *in vivo* (39–41). This is especially true for proteins interfering with the host immune system, such as ROPs and GRAs (42), or that are involved in specific metabolic pathways (43, 44). To better define the Slp requirement during a natural infection, 100 or 1,000 tachyzoites of Δ*ku80* or Slp-KO parasites were inoculated intraperitoneally into immunocompetent BALB/c mice, and their survival was monitored daily (Fig. 6A and B). While 100% of the mice succumbed to an infection with Δ*ku80* within 8 to 12 days, most mice survived an infection with Slp-KO parasites. The seroconversion of the infected mice was systematically verified by Western blotting, and the seronegative mice were excluded from the analysis (Fig. S7). Importantly, the virulence was largely restored in the Compl strain. At higher parasite doses, the mice were equally susceptible to the infection (Fig. 6C). These results highlight a role for TgSlp in the maintenance of T. gondii virulence.

10.1128/msphere.00350-22.7 FIG S7 Mouse seroconversion following injection of 100 tachyzoites was verified by immune reactivity of the sera from infected mice (numbered M1 to M7) with tachyzoite extracts by Western blotting. While serum from noninfected mice showed no reactivity (left panel, negative control), the sera from all but one tested mouse (seronegative) reacted with tachyzoite lysates. The complex profile of bands detected by the mouse sera indicates that the mice were successfully infected with the parasite. Seronegative mice were excluded from subsequent analyses. Download FIG S7, TIF file, 0.9 MB. Copyright © 2022 Najm et al. 2022 Najm et al. https://creativecommons.org/licenses/by/4.0/ This content is distributed under the terms of the Creative Commons Attribution 4.0 International license.

### The role of Slp in T. gondii virulence is independent of its localization in the PV.

We previously demonstrated that TgSlp is an ER-resident protein and that its secretion in the PV likely represents mistargeting due to its C-terminal tagging. To assess T. gondii virulence when Slp is located both at the ER and in dense granules or restricted to the ER only, we infected mice with both Slp-tagged lines (Slp-HA_3_Ct and Slp-HA_3_). Both lines showed similar virulence, further supporting that the function of Slp is not linked to its association with dense granules and release in the PV (Fig. 6D).

## DISCUSSION

Here, we have characterized a phosphatase of T. gondii showing similarity to bacterial phosphatase. Our data demonstrate that Slp is an active phosphatase primarily located in the ER of the parasite. Although not essential for parasite propagation in human fibroblasts *in vitro*, the Slp-KO line is slightly affected in its invasive capacity. More importantly, our data uncovered an important function for this bacterial-like phosphatase *in vivo* that may be of relevance in the context of a natural infection.

We started our characterization of Slp phosphatase by investigating its subcellular localization. The introduction of a C-terminal epitope (Slp-HA_3_Ct) allowed the localization of the protein at the nucleus periphery as well as in the PV lumen, where it colocalized with the dense granule protein GRA2. These observations were concordant with those of a previous study that similarly reported partial colocalization of *Tg*Slp with GRA12 in the PV of intracellular parasites (29). However, it did not fit well with the ER localization of the protein by HyperLOPIT (33). To address this discrepancy, we introduced a tag before the *Tg*Slp putative ER retention signal (Slp-HA_3_). In this parasite line, *Tg*Slp was exclusively detected in the ER, and importantly, its expression pattern oscillated along the cell cycle, concordant with the transcriptomic data (32). Therefore, our results demonstrated that a free C terminus is important for the proper localization and timing of expression of the protein. The C-terminal GDEL sequence of *Tg*Slp closely resembles the canonical KDEL motif for soluble ER protein retrieval in mammalian cells (45). Since its discovery, many variants of this motif have now been described, especially in the first position (46). Mutagenesis studies will help verify the importance of this motif for *Tg*Slp subcellular localization.

In our hands, Slp-KO parasites exhibited a minor impairment during invasion of human foreskin fibroblasts (HFFs) *in vitro*, which was reverted by complementation. The similar localization of T. gondii Slp at the ER, with regard to *Pb*Slp1 for which a role in micronemes biogenesis had been suggested, prompted us to investigate carefully the formation of micronemes (and other secretory organelles) and microneme secretion in Slp-KO parasites. The assembly of micronemes and rhoptries takes place *de novo* in the emerging daughter cells and relies on an unconventional trafficking process based on exocytic and endocytic machineries, during which protein synthesis and transport are intimately linked to organelle biogenesis (47–50). The biosynthesis of MIC proteins begins in the classical early secretory pathway, where they are targeted to the ER before being routed to the Golgi apparatus. From there, they are sorted into endosomal-like compartments, relying on the recognition of tyrosine-based motifs in their cytoplasmic tails (51), before eventually reaching immature promicronemes and then micronemes. Given its ER localization, Slp may well play a role in vesicular trafficking for secretory organelle assembly. However, we did not observe any ultrastructural defect in the biogenesis, positioning, nor secretion capacity of the micronemes in Slp-KO parasites. As *Plasmodium* merozoites and *Toxoplasma* tachyzoites both harbor many fewer micronemes than other parasite stages (ookinetes for *Plasmodium*, sporozoites and bradyzoites for *Toxoplasma*) (52), we did not exclude a role for Slp in microneme biogenesis and trafficking in bradyzoites or feline merozoites, during which *slp* is transcribed at a higher rate than in tachyzoites (53–55).

In contrast to the mild effect on the invasive capacity of the parasite *in vitro*, the loss of Slp led to a 3-log reduction of T. gondii virulence in mice. This result diverged from that reported by Liang et al., in which the knockout of *slp* did not affect the virulence of type I or type II parasites *in vivo* (29). The reasons for the discrepancies between the two studies are still unclear. Yet, the fact that our phenotype can be complemented by restoring the *slp* wild-type locus clearly suggests that the observed defects are linked to the loss of the *slp* gene itself. Many parasitic effectors secreted from dense granules and rhoptries hijack the host cell response to infection. Concordantly, the genetic disruption of these effectors usually compromises the strain virulence *in vivo*, while parasite growth remains unaffected *in vitro* (42). Here, we showed that when Slp was restricted to an ER localization and no longer present in the dense granules and secreted in the PV, the parasite maintained its virulence *in vivo*. This suggested that Slp function is not linked to its secretion from dense granules and therefore should not be considered a *bona fide* secreted effector of T. gondii.

Based on its cellular localization in the parasite ER, we hypothesized that Slp might be required for the parasite to cope with ER stress. In mammalian cells, ER stress triggers the unfolded protein response (UPR), a mechanism that enables cells to restore ER homeostasis and thus ensures cell survival. In mammals, three ER transmembrane proteins act as molecular sensors of the accumulation of misfolded proteins, namely, PERK, ATF6, and IRE1 (56). T. gondii parasites lack ATF6 and IRE1 but encode a UPR sensor related to PERK (TgIF2K-A) that is localized to the ER (57–59). In mammals, PERK represses global protein synthesis by phosphorylating the eukaryotic translation initiation factor eIF2α on residue serine 51 (59, 60). PERK optimal activity is regulated by *trans*-autophosphorylation (61, 62) and by the activity of an ER-located phosphatase called tyrosine phosphatase 1B (PTP-1B) (63–65). PTP-1B plays a pleiotropic role not only in the regulation of metabolic homeostasis, but also in ER stress, as its deficiency leads to an increase in PERK-eIF2α signaling (66, 67). Interestingly, T. gondii parasites that are unable to phosphorylate eIF2α, due to the replacement of the serine residue 71 by an alanine (equivalent to Ser-51 in mammals), are less viable than wild type after incubation in the extracellular milieu, suggesting that phosphorylation of eIF2α takes place at the time of egress and promotes parasite survival until the next reinvasion (68). *Tg*Slp protein shares similar features with mammalian PTP-1B, including its ER localization and possibly its tyrosine phosphatase activity. Therefore, if Slp does indeed regulate the ER stress via TgIF2K-A/eIF2α when the tachyzoites become extracellular, the much more stressful environment encountered by the parasite *in vivo* compared to *in vitro* might account for the difference of phenotype observed between the two growth conditions. Whether Slp participates in the regulation of the UPR remains to be explored.

Overall, our data identified Shelph as an important phosphatase for *Toxoplasma* virulence. As this family of bacterial-like phosphatases are absent from human genomes, it will be relevant to explore their functions in other stages of the parasite life cycle, as they may represent future candidates for therapeutic interventions.

## MATERIALS AND METHODS

### Parasite strains, cell culture, and transfection.

All T. gondii parasite strains were generated from T. gondii RH-Δ*ku80* strain (69), abbreviated Δ*ku80* throughout the study. Parasites were maintained and passaged *in vitro* on human foreskin fibroblasts (HFFs; ATCC CRL 1634) in Dulbecco’s modified Eagle’s medium (Gibco BRL) supplemented with 5% fetal calf serum (FCS), 2 mM glutamine, and penicillin-streptomycin (Gibco) at 100 μg/mL, 37°C, 5% CO_2_. Freshly egressed parasites were transfected by electroporation as described previously (70). Transgenic parasites were selected with 2 μM pyrimethamine for pYFP-LIC-DHFR integration, 25 μg/mL mycophenolic acid and 50 μg/mL xanthine for *HXGPRT* cassette selection, 5 μM fluorodeoxyuridine for pUPRT-MIC2-Gluc, and 30 μg/mL phleomycin for PSBLE35 cosmid. All the transgenic lines obtained were verified by diagnostic PCR and sequencing.

### Molecular biology.

For cloning, PCRs were performed using Q5 high-fidelity DNA polymerase (New England Biolabs), and all the fragments were sequenced before further use. PCRs amplicons directly used for parasite transfections were amplified with KOD polymerase (Novagen). Integrative PCRs were done using the GoTaq master mix (Promega). RNA extraction and reverse transcription (RT) were performed using the NucleoSpin RNA kit (Macherey-Nagel) and the Superscript first-strand synthesis system (Invitrogen), respectively, according to the manufacturers’ instructions. *zfp2* and *slp* cDNAs were amplified using primers ML2877/ML2878 and ML1279/ML1280, respectively. All the primers used in this study are listed in Table S1 in the supplemental material.

10.1128/msphere.00350-22.8 TABLE S1 List of primers used in this study. Download Table S1, DOCX file, 0.02 MB. Copyright © 2022 Najm et al. 2022 Najm et al. https://creativecommons.org/licenses/by/4.0/ This content is distributed under the terms of the Creative Commons Attribution 4.0 International license.

### Plasmids.

**(i) Slp-HA_3_Ct.** Slp-HA_3_Ct parasites were obtained by fusing a triple HA tag (HA_3_) at the 3′ end of the *slp* gene using the pYFP-LIC-DHFR vector (69). Briefly, a fragment of 1,260 bp of *slp* was amplified using primers ML1279 and ML1280, containing complementary LIC sequences, and inserted by ligation-independent cloning in pYFP-LIC-DHFR. The resulting plasmid was linearized with EcoRV before transfection of RHΔ*ku80* parasites. Single homologous recombination allowed the integration of the whole plasmid, generating the Slp-HA_3_Ct line.

**(ii) Slp-HA_3_.** Three overlapping PCR fragments corresponding to Shelph amino acids 335 to 407, HA_3_ tag, and Shelph amino acids 408 to 421 with 194 bp of the 3′-UTR were amplified using primers MLa140/MLa141, MLa142/MLa171, and MLa144/MLa145, respectively. A long PCR fragment of 540 bp was then obtained with the KOD DNA polymerase by mixing the 3 PCR products and primers MLa140 and MLa145. In addition, a guide RNA targeting amino acids 396 to 402 (gRNA MLa95bis-96 bis) was cloned into the BsaI restriction site of the pU6-Universal plasmid (71).

**(iii) Slp-KO and complementation.** To knock out the *slp* locus in the Slp-HA_3_Ct background, we used CRISPR-Cas9 technology. The guide RNA was obtained by annealing of primers MLa97 and MLa98, followed by BsaI cloning in the pU6-Universal vector. The donor DNA consisted of the PCR amplification of the *HXGPRT* cassette flanked on both sides by 30-bp of homology to the *slp* locus with primers MLa93 and MLa100. Thirty micrograms of pU6-gRNA was cotransfected with 50 μL of PCR product in the Slp-HA_3_Ct line. Transgenic parasites were selected with mycophenolic acid and xanthine.

Complementation of Slp-KO parasites was obtained by transfection of PSBLE95 cosmid, followed by selection with phleomycin and cloning by limiting dilution.

**(iv) pUPRT-MIC2-Gluc.** A 4.7-kb fragment comprising the MIC2 promoter, the *mic2* gene fused to Gaussia luciferase, and the *mic2* 3′-UTR was amplified using primers ML4941 and ML4942 from pMIC2-GLuc-cMyc (6) and cloned by in-fusion HD (TaKaRa) in the pUPRT-TUB-TgRASP2-Ty plasmid (72), opened with NotI/AvrII restriction sites. The resulting pUPRT-MIC2-Gluc vector was linearized with NdeI prior to transfection and then cotransfected along with pU6-UPRT plasmid encoding a guide RNA targeting the *uprt* locus (70).

**(v) pGex4T3-Slp.** Slp protein was expressed as an N-terminal GST-fusion protein. The sequence corresponding to amino acids 84 to 450 of Slp-HA_3_ was amplified by PCR from the genomic DNA of Slp-HA_3_ parasites using primers MLa383 and MLa384 and then cloned in-frame with GST into XhoI and EcoRI restriction sites of the pGex4T3 vector (GE Healthcare Life Sciences). The resulting vector was transformed into the BL21-DE3(pLysS) bacterial strain (Novagen).

### Recombinant protein purification and phosphatase assay.

E. coli cells expressing either the control recombinant GST (rGST) protein or GST-Slp-HA_3_ (rSlp) were induced during exponential growth with 0.5 mM isopropyl-β-d-thiogalactopyranoside at 37°C for 3 h, and the bacterial cell pellets were lysed using a French press. The soluble recombinants recovered from the supernatants were affinity purified on glutathione-agarose beads (Sigma). Following washes with PBS–0.1% Triton, proteins were eluted in 20 mM reduced glutathione and dialyzed in 20 mM Tris-HCl (pH 8.0), 400 mM NaCl, 0.5 mM MnCl_2_ overnight at 4°C.

Phosphatase assays were performed in 100 μL reaction buffer (20 mM Tris-HCl [pH 8.0], 400 mM NaCl, 0.5 mM MnCl_2_, 1 mM dithiothreitol) at 37°C for 30 min, with 5 mM pNPP (Bio Basic Inc.) and 0.5 μg of protein unless otherwise stated. The absorbance of pNP was read at 405 nm using a Tecan spectrophotometer. To calculate the quantities of pNP released from the reaction, a molar extinction coefficient of 18,000 M^−1 ^cm^−1^ was used.

### Western blotting.

Freshly egressed parasites (5 × 10^6^) were lysed in Laemmli buffer and boiled for 5 min. Proteins were separated on an 8 to 12% acrylamide-bisacrylamide gel by SDS-PAGE and transferred onto nitrocellulose membrane. Following blocking in PBS–5% milk for 30 min, the membrane was probed with primary antibodies diluted in PBS–5% milk for 2 h and revealed with secondary antibodies conjugated to alkaline phosphatase (Promega) for 1 h. Proteins were visualized by the addition of color development substrates (nitroblue tetrazolium–5-bromo-4-chloro-3-indolylphosphate; Promega). The antibody dilutions were as follows: rat anti-HA, 1/1,000; mouse anti-SAG1, 1/2,000.

### Immunofluorescence assay.

Following fixation in 4% paraformaldehyde (PAF) for 30 min, cells were permeabilized using PBS–0.1% Triton or PBS-saponin (invasion) for 10 min, then saturated for 30 min in PBS–10% FCS. Primary antibodies diluted in PBS–2% FCS were incubated for 1 h. Following 3 washes in PBS, Alexa-coupled secondary antibodies diluted in PBS–2% FCS were incubated for 1 h. Nuclei were stained with Hoechst for 5 min. Cells were washed 3 times in PBS before mounting. Samples were observed on a Zeiss Axio Imager microscope at the Montpellier RIO Imaging Facility and processed using ZEN software. The antibody dilutions were as follows: rabbit anti-GAP45, 1/5,000 (73); rat anti-HA 3F10, 1/100 (Roche catalog number 11867423001); rabbit anti-AMA1, 1/5,000 (74); mouse anti-MIC3, 1/300 (75); mouse anti-ROP2-4, 1/100 (76); mouse anti-GRA1, 1/300 (77); rabbit anti-GRA2, 1/500 (78); rabbit anti-GRA3, 1/500 (79); mouse anti-SAG1, 1/2,000 (80).

### Invasion, replication, and egress assays.

Freshly egressed parasites (5 × 10^6^ for invasion, 5 × 10^5^ for replication and egress) were inoculated on HFFs grown on coverslips for 5 min (invasion) or 1 h (replication, egress) before extensive washes in Hank’s balanced salt solution (Gibco BRL). Invasion was stopped by fixation in PAF and further processed for dual-labeling IFA using anti-SAG1 and anti-ROP1 antibodies. Parasite multiplication was allowed to take place for another 24 h (replication) before fixation in PAF and further processing by IFA using anti-SAG1 antibodies. For egress, parasites at 30 h postinvasion were treated with dimethyl sulfoxide (control) or 3 μM A23187 for 8 min at 37°C, prior to fixation in PAF and dual labeling using anti-GRA3 and anti-GAP45 antibodies.

### Gliding motility assay.

Freshly egressed parasites (1 × 10^6^) were resuspended in 300 μL of gliding buffer (155 mM NaCl, 3 mM KCl, 2 mM CaCl_2_, 1 mM MgCl_2_, 3 mM NaH_2_PO_4_, 10 mM HEPES, 10 mM glucose). A 100-μL aliquot of this suspension was deposited on a poly-l-lysine-coated slide in a well delineated with a hydrophobic pen. Parasites were allowed to glide for 15 min at 37°C, before fixation in 4% PAF. The slides were further processed by IFA, except that the permeabilization step was omitted. Trails were stained with an anti-SAG1 antibody. Images were processed with ImageJ using the NeuronJ plug-in.

### Microneme secretion assay.

The microneme secretion assay was performed as previously described (6). Briefly, confluent HFF monolayers were inoculated with Δ*ku80*/MIC2Gluc or Slp-KO/MIC2Gluc tachyzoites for 48 h to attain large vacuoles with minimal cell lysis. Infected HFFs were then scraped, shifted to an 18°C water bath to prevent premature microneme secretion, and passed through a 25-gauge needle to release intracellular parasites. After centrifugation (400 × *g*) for 10 min at 18°C, the parasites were resuspended in EC buffer (5 mM KCl, 142 mM NaCl, 1 mM MgCl_2_, 1.8 mM CaCl_2_, 5.6 mM d-glucose, 25 mM HEPES; pH 7.4). To stimulate microneme secretion, 50 μL of mock or 50 μL of 1 mM propranolol was added to 50 μL EC buffer containing 5 × 10^5^ parasites in a 96-well V-bottom plate and incubated in a water bath at 37°C for 10 min. The stimulation was stopped by shifting the plate to ice for 5 min. The supernatant was collected following centrifugation (1,200 × *g*) for 10 min at 4°C and used to measure the released MIC2-Gluc. The BioLux Gaussia Flex luciferase kit (Pierce) was used according to the manufacturer’s instructions. The relative luminescence (RLU) was read on a Centro LB960 luminometer (Berthold Technologies).

### Transmission electron microscopy.

HFF monolayers were infected with Δ*ku80* or Slp-KO parasites and grown for an extra 24 h. They were fixed with 2.5% glutaraldehyde in cacodylate buffer, 0.1 M, pH 7.4. Coverslips were then processed using a Pelco Biowave pro+ (Ted Pella). Briefly, samples were postfixed in 1% OsO_4_ and 2% uranyl acetate, dehydrated in an acetonitrile series, and embedded in Epon 118 using the following parameters: glutaraldehyde (150 W on/off/on 1-min cycles); two buffer washes (40 s, 150 W); OsO_4_ (150 W on/off/on/off/on 1-min cycles); two water washes (40 s, 150 W); uranyl acetate (100 W on/off/on 1-min cycles); dehydration (40 s, 150 W); and resin infiltration (350 W, 3-min cycles). Fixation and infiltration steps were performed under vacuum. Polymerization was performed at 60°C for 48 h. Ultrathin sections at 70 nM were cut with a Leica UC7 ultramicrotome, counterstained with uranyl acetate and lead citrate, and observed in a Jeol 1400+ transmission electron microscope from the MEA Montpellier Electron Microscopy Platform. All chemicals were from Electron Microscopy Sciences, and solvents were from Sigma.

### Ethics statement.

All mice protocols were approved by the Institutional Animal Care and Utilization Committee (IACUC) of the American University of Beirut (AUB) (permit number 18-02-461). All animals were housed in a specific-pathogen-free facility with a 12-h on/off light cycle. Humane endpoints were fully respected as per AUB IACUC, following Association for Assessment and Accreditation of Laboratory Animal Care International guidelines and the NRC Guide of animal care use book (Guide, NRC 2011). Mice were monitored on a daily basis. To verify the acute phase of the infection, blood was withdrawn following deep anesthesia with isoflurane by inhalation. Mice were sacrificed if any abnormal ethical features were noticed. Animals were deeply anesthetized before cervical dislocation.

### Mouse infection.

Eight- to 10-week-old immunocompetent female BALB/c mice were intraperitoneally injected with Δ*ku80*, Slp-KO, Compl, Slp-HA_3_Ct, or Slp-HA_3_ lines. To assess the virulence *in vivo*, freshly harvested tachyzoites (10^2^, 10^3^, or >10^4^) were intraperitoneally injected into mice. The number of mice used per condition and the number of independent experiments are stated in the legend of each graph. Invasiveness of the parasites was evaluated by simultaneous plaque assay of a similar dose of parasites on HFFs. Immunoreactivity against tachyzoite antigens with serum IgG from mammals having acute toxoplasmosis was used as an indicator of infection (81). For this, on day 6 postinfection, blood samples from each infected mouse were withdrawn using retroorbital puncture. Acute toxoplasmosis was verified by immune reactivity of infected mice with tachyzoite extracts by Western blotting. The seronegative mice were excluded from the analyses. Following mouse injections, their survival was daily monitored until their death, the endpoint of all experiments.
