# Supplementary figures and images for: Toxoplasma Shelph, a Phosphatase Located in the Parasite Endoplasmic Reticulum, Is Required for Parasite Virulence
Source: mSphere. 2022 Nov 3;7(6):e00350-22. doi: 10.1128/msphere.00350-22 (PMC9769683; doi:10.1128/msphere.00350-22)

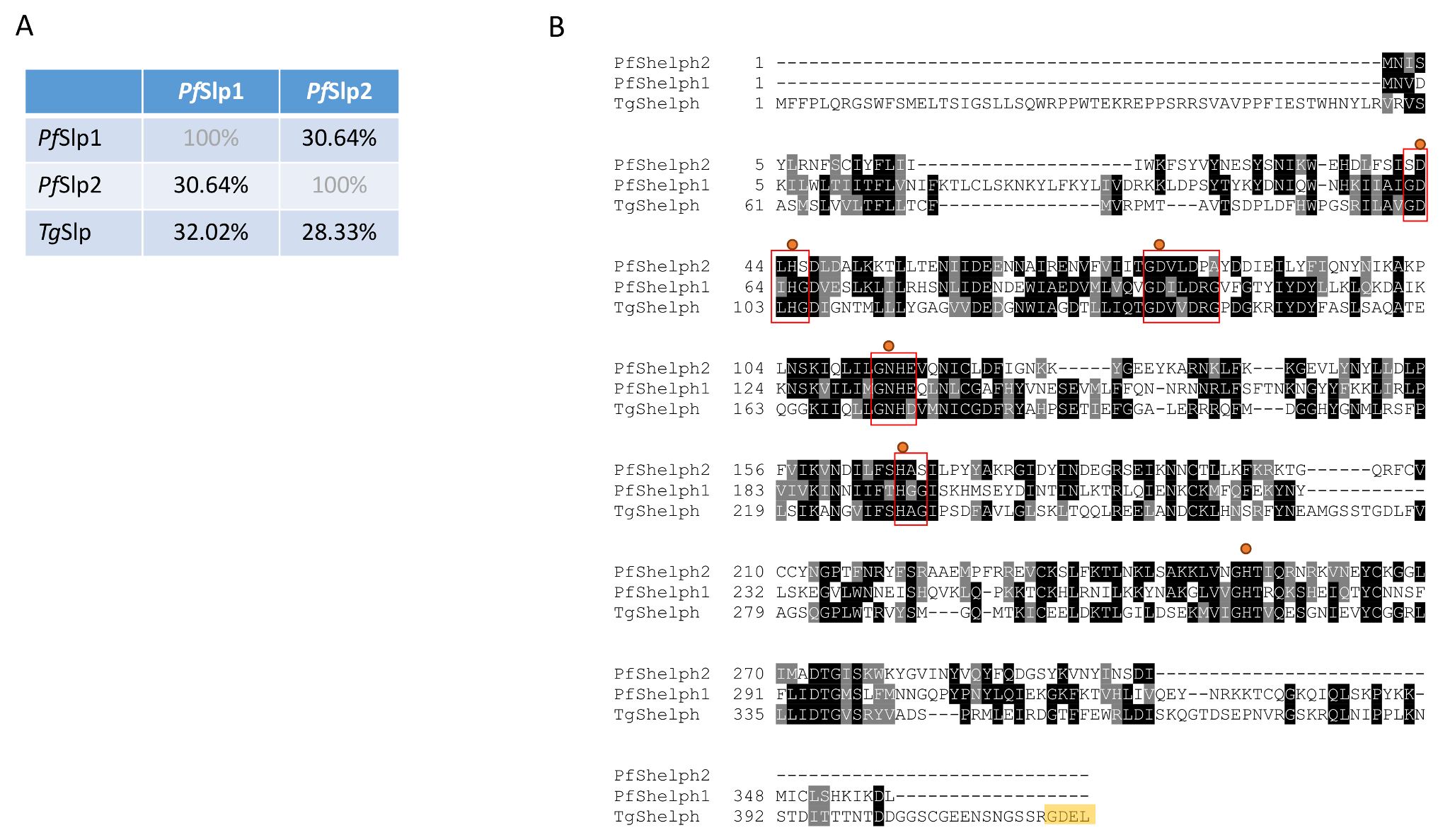

Supplement: FIG S1 [file msphere.00350-22-s0001.tif]

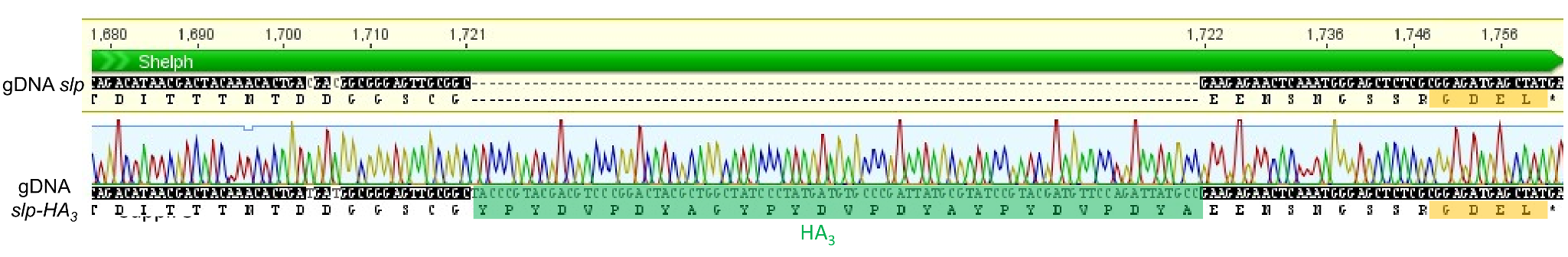

Supplement: FIG S2 [file msphere.00350-22-s0002.tif]

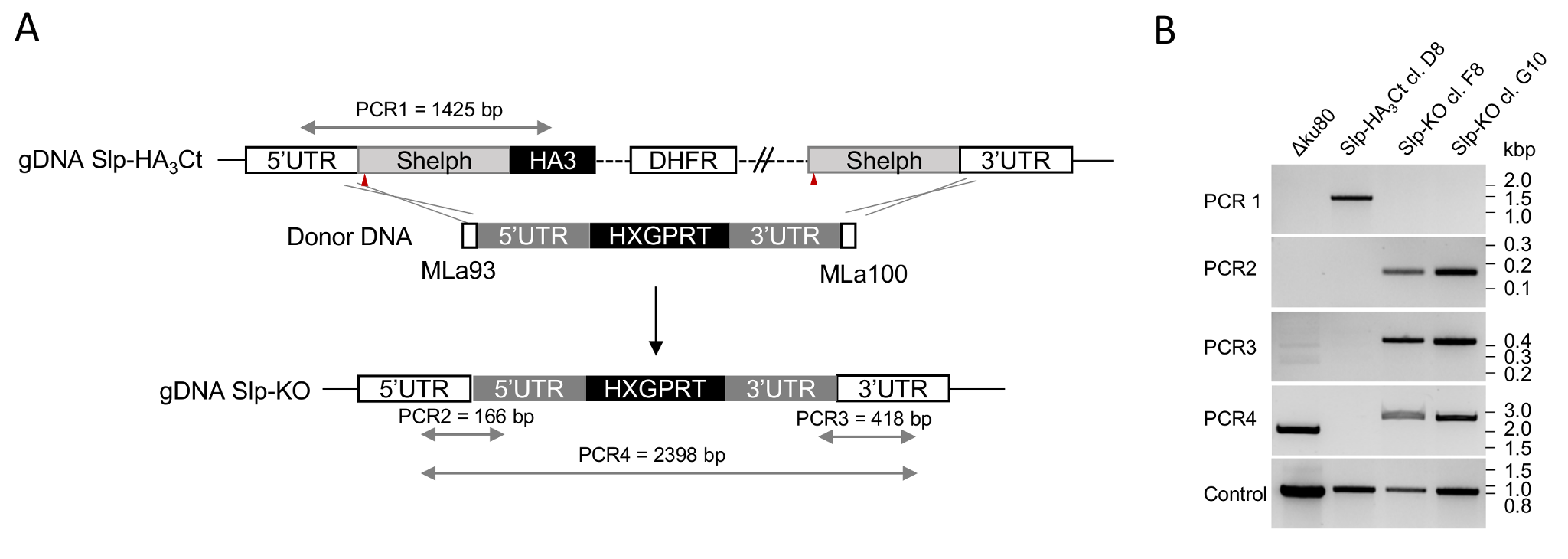

Supplement: FIG S3 [file msphere.00350-22-s0003.tif]

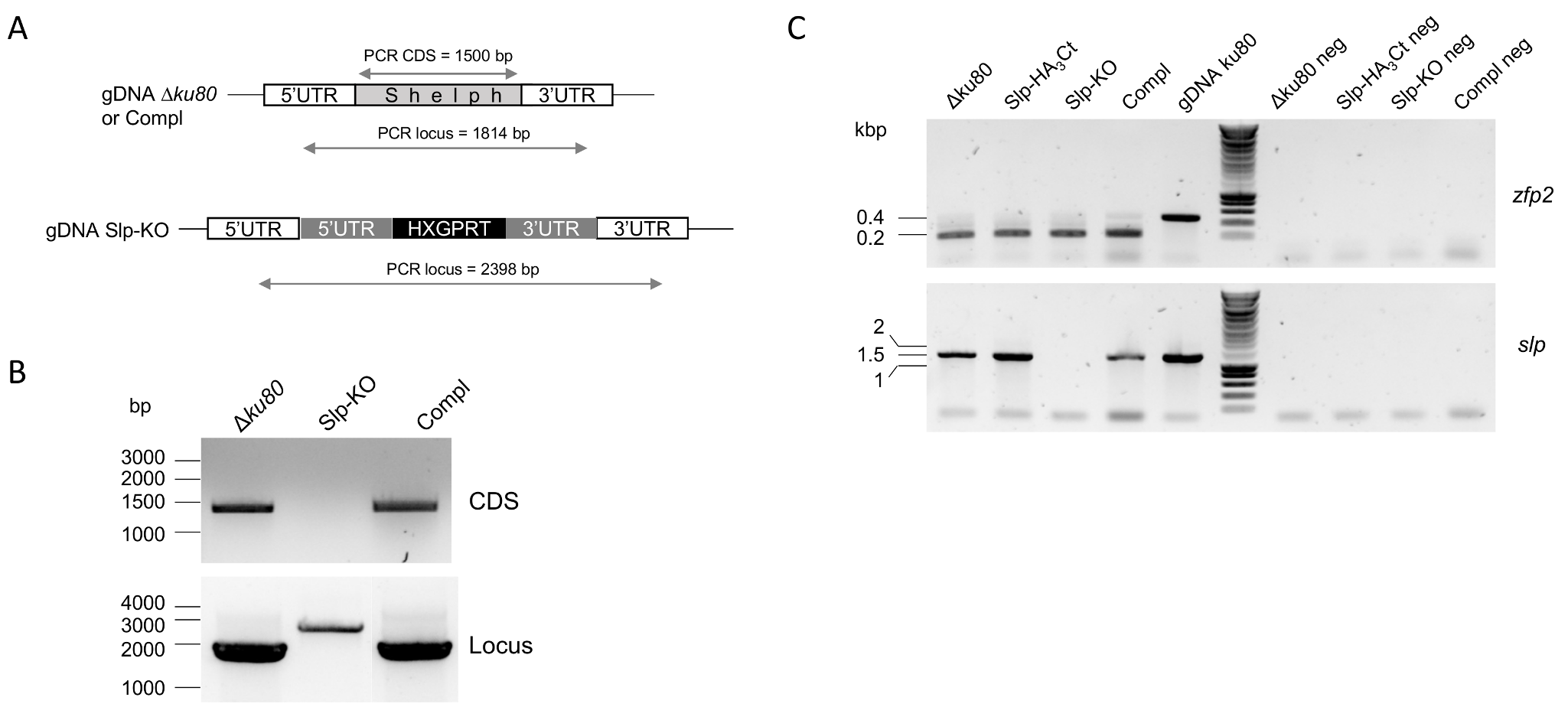

Supplement: FIG S4 [file msphere.00350-22-s0004.tif]

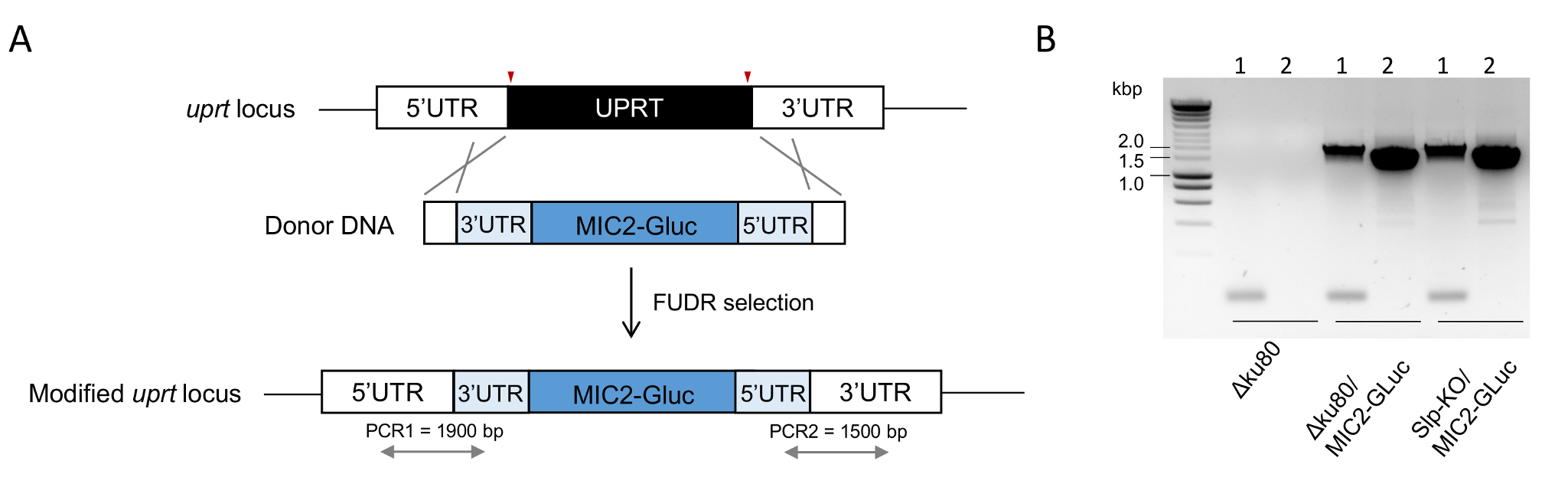

Supplement: FIG S5 [file msphere.00350-22-s0005.tif]

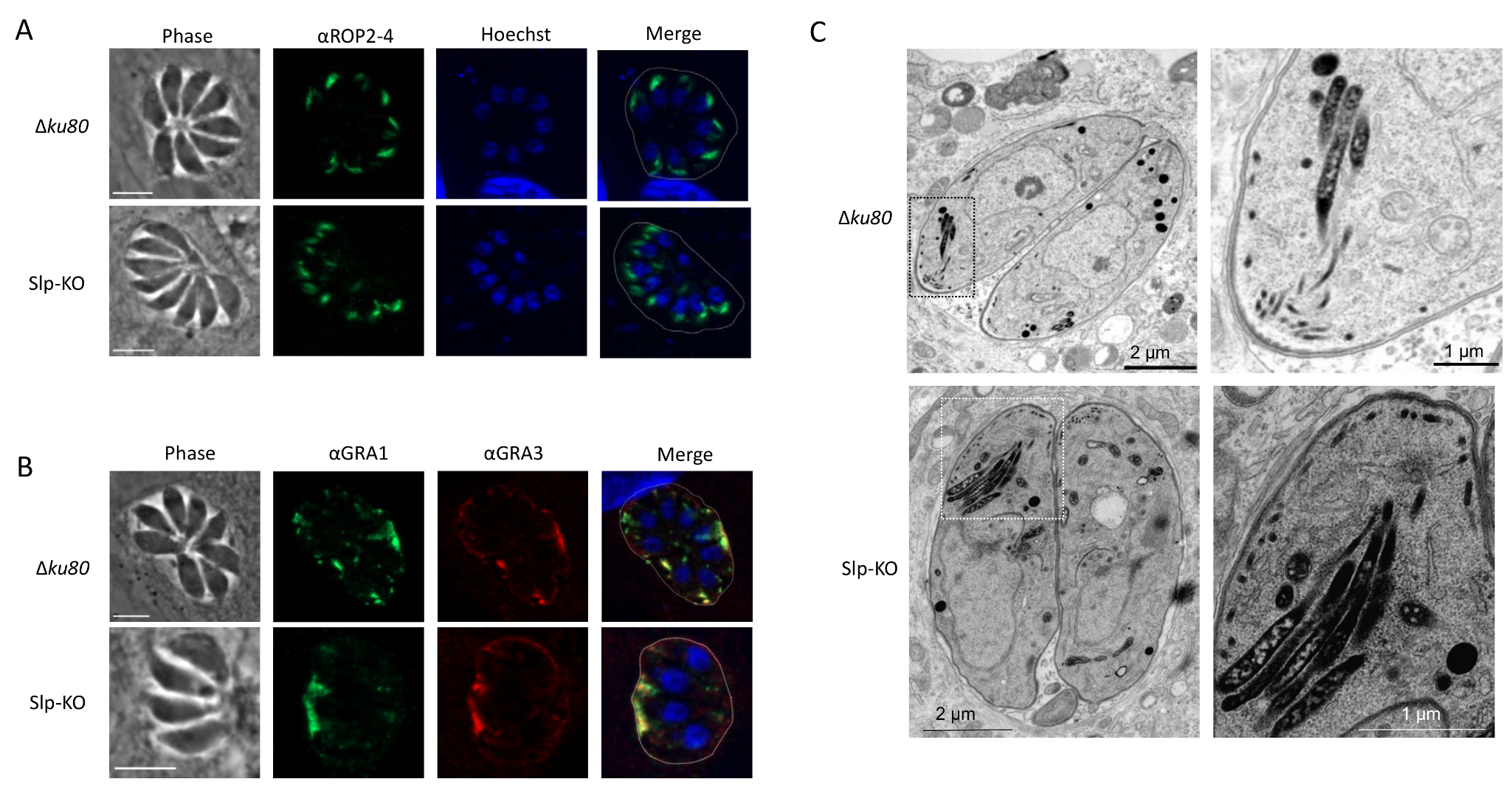

Supplement: FIG S6 [file msphere.00350-22-s0006.tif]

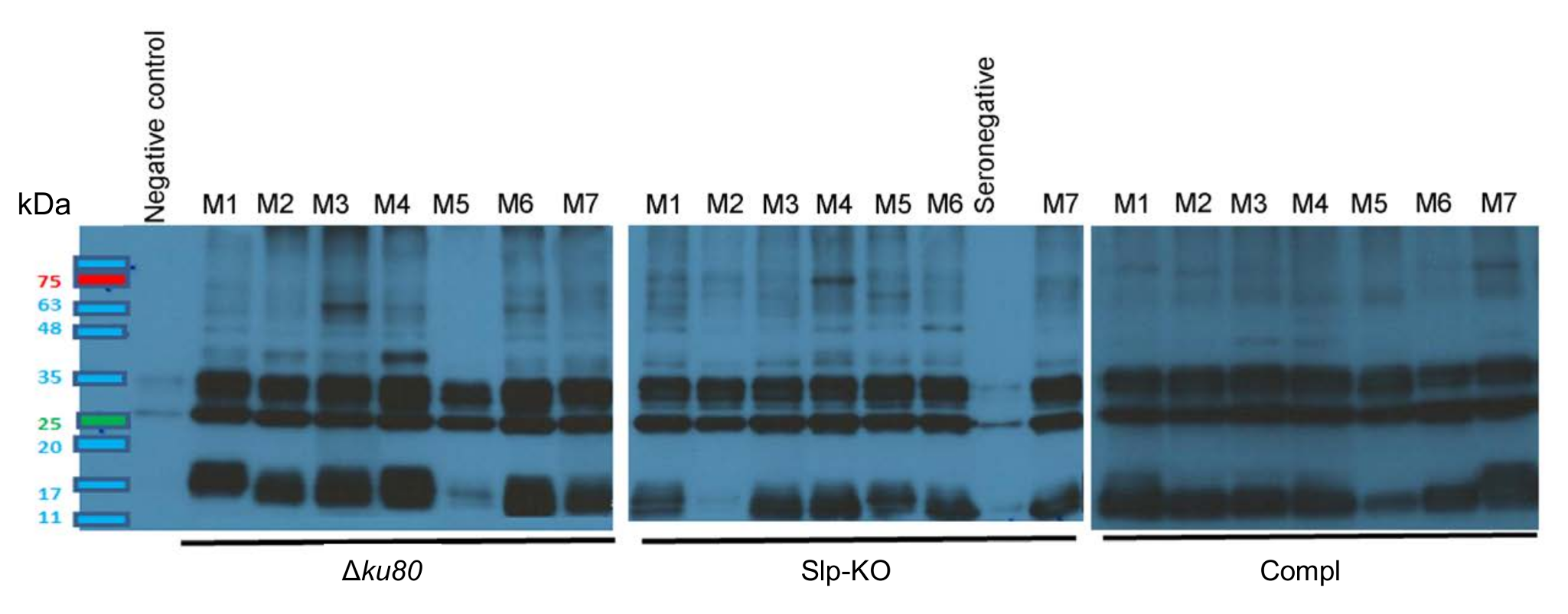

Supplement: FIG S7 [file msphere.00350-22-s0007.tif]
