# Supplementary material for: Toxoplasma Shelph, a Phosphatase Located in the Parasite Endoplasmic Reticulum, Is Required for Parasite Virulence
Source: mSphere. 2022 Nov 3;7(6):e00350-22. doi: 10.1128/msphere.00350-22 (PMC9769683; doi:10.1128/msphere.00350-22)
Supplement: TABLE S1 [file msphere.00350-22-s0008.docx]

**Supplemental Table 1 : list of primers used in this study**

| Primer name | Orientation | Sequence (5’-3’) | Purpose |
| --- | --- | --- | --- |
| ML875 | Fwd | CACACGAAACCACAGGAAAGG | PCR control in Fig.1B; PCR control in Suppl. Fig.3B |
| ML876 | Rev | CTTTTGCCAGGTGTTTCGGTAG | PCR control in Fig.1B; PCR control in Suppl. Fig.3B |
| ML1042 | Rev | GCCGACAGGACGCTACTGG | PCR2 in Suppl. Fig.3A-B |
| ML1043 | Fwd | CAGTGACACCGCGGTGGAGG | PCR3 in Suppl. Fig.3A-B |
| ML1279 | Fwd | TACTTCCAATCCAATTTAATGCATGTTTTTCCCGCTCCAACGTGG | Cloning slp in pYFP-LIC-DHFR; PCR CDS and RT-PCR *slp* in Suppl Fig. 4A-C |
| ML1280 | Rev | TCCTCCACTTCCAATTTTAGCTAGCTCATCTCCGCGAGAGCTCC | Cloning slp in pYFP-LIC-DHFR; PCR CDS and RT-PCR *slp* in Suppl Fig. 4A-C |
| ML1422 | Fwd | CGGAGCGCTATCTCGC | PCR1 in Fig.1A-B, PCR1 in Suppl Fig. 3A-B |
| ML1476 | Rev | CAGCGTAGTCCGGGACGTCGTAC | PCR1 in Fig. 1A-B, PCR1 in Fig. 2A-B, PCR1 in Suppl Fig. 3A-B |
| ML2877 | Fwd | AATCATCTGAGCGAGCTGTC | RT-PCR *zfp2* |
| ML2878 | Rev | TTGAACCGTGTGCATGGTG | RT-PCR *zfp2* |
| ML4941 | Fwd | CAGCCTCACGTTAACGCGGCCGCGTGTGGAATTGTGAGCGGAT | Cloning MIC2-Gluc in pUPRT-TUB-TgRASP2-Ty |
| ML4942 | Rev | GTTCGTGTGGACCTCCCTAGGACCGAGCAGATTGGCGAAC | Cloning MIC2-Gluc in pUPRT-TUB-TgRASP2-Ty |
| ML5076 | Fwd | CTTGTCTGACGAACCTCTCG | PCR1 in Suppl. Fig.5A-B |
| ML2866 | Rev | CGTTCATTCGAAACAAGGAC | PCR1 in Suppl. Fig.5A-B |
| ML5077 | Rev | CAATCTCACTGGCACGGA | PCR2 in Suppl. Fig.5A-B |
| ML4780 | Fwd | CCGAGGAAATGCAGTGTAGC | PCR2 in Suppl. Fig.5A-B |
|  |  |  |  |
| MLa93 | Fwd | AACGGCAGGCTATCAGTTTCTAACGCAACCGATCAGCACGAAACCTTGCA | Donor DNA for Slp-KO line |
| MLa95bis | Fwd | AAGTTGACGACTACAAACACTGACGAG | gRNA for Slp-HA_3_ line |
| MLa96bis | Rev | AAAACTCGTCAGTGTTTGTAGTCGTCA | gRNA for Slp-HA_3_ line |
| MLa97 | Fwd | AAGTTGTCCATGGAGCTCACATCCATG | gRNA for Slp-KO line |
| MLa98 | Rev | AAAACATGGATGTGAGCTCCATGGACA | gRNA for Slp-KO line |
| MLa100 | Rev | CTGTCCTTTTGAAACTCTGCTAGCACAGGGCCTCCACCGCGGTGTCACT | Donor DNA for Slp-KO line |
| MLa102 | Rev | CTGCTCTGGTAGGTGGTTCG | PCR3 and PCR4 in Suppl. Fig.3A-B; PCR locus in Suppl. Fig. 4B |
| MLa140 | Fwd | CCTCCTGATCGACACAGGAG | Donor DNA for Slp-HA_3_ line |
| MLa141 | Rev | GCCGCAACTCCCGCCATCATCGGTGTTTGTAGTCGTT | Donor DNA for Slp-HA_3_ line |
| MLa142 | Fwd | GCGGGAGTTGCGGCTACCCGTACGACGTCCC | Donor DNA for Slp-HA_3_ line; PCR2 in Fig. 2A-B |
| MLa144 | Fwd | GAAGAGAACTCAAATGGGAGCTC | Donor DNA for Slp-HA_3_ line |
| MLa145 | Rev | GGAGGGCACACATATCTGG | Donor DNA for Slp-HA_3_ line |
| MLa170 | Fwd | ATTCCATGGGGCAAATGACG | PCR1 and PCR3 in Fig. 2A-B |
| MLa171 | Rev | CCATTTGAGTTCTCTTCGGCATAATCTGGAACATCGTACGGA | Donor DNA for Slp-HA_3_ line |
| MLa310 | Rev | CAAAATTCTCACACGTTACGTCC | PCR2 and PCR3 in Fig. 2A-B |
| MLa383 | Fwd | CGCGAATTCCACATCTGATCCCCTTGACTTTC | Cloning rSlp in pGex4T3 |
| MLa384 | Rev | CGCCTCGAGTCATAGCTCATCTCCGCGAG | Cloning rSlp in pGex4T3 |
